# Upregulation of Coagulation Factor VIII and Fibrinogen After Pulmonary Endarterectomy in Patients with Chronic Thromboembolic Pulmonary Hypertension

**DOI:** 10.1177/10760296231158369

**Published:** 2023-03-08

**Authors:** Lasse Myllylahti, Jussi Ropponen, Mikko Lax, Riitta Lassila, Antti I. Nykänen

**Affiliations:** 1 Division of Internal Medicine, Department of Internal Medicine and Rehabilitation, Helsinki University Hospital, Helsinki, Finland; 2 Department of Cardiac Surgery, Heart and Lung Center, Helsinki University Hospital and University of Helsinki, Helsinki, Finland; 3 Division of Anesthesiology, Department of Anesthesiology, Intensive Care and Pain Medicine, Helsinki University Hospital, Helsinki, Finland; 4 Unit of Coagulation Disorders, Department of Hematology, Comprehensive Cancer Center, Helsinki University Hospital, and Research Program Unit in Systems Oncology, 3835 University of Helsinki, Helsinki, Finland

**Keywords:** chronic thromboembolic pulmonary hypertension, pulmonary endarterectomy, anticoagulation, LMWH, coagulation biomarkers, pulmonary embolism

## Abstract

**Objectives:**

Chronic thromboembolic pulmonary hypertension (CTEPH) is associated with thrombotic states including elevated coagulation factor VIII (FVIII). Pulmonary endarterectomy (PEA) is the main treatment for CTEPH, and efficient anticoagulation is essential to prevent thromboembolism recurrence after surgery. We aimed to characterize longitudinal changes in FVIII and other coagulation biomarkers after PEA.

**Methods:**

Coagulation biomarker levels were measured at baseline and up to 12 months after operation in 17 consecutive patients with PEA. Temporal patterns of coagulation biomarkers, and correlation of FVIII with other coagulation biomarkers, were analyzed.

**Results:**

Baseline FVIII levels were elevated in 71% of the patients (mean 216 ± 67 IU/dl). FVIII doubled 7 days after PEA, peaking at 471 ± 87 IU/dl, and gradually returned to respective baseline levels within 3 months. Postoperative fibrinogen levels were also elevated. Antithrombin decreased at 1 to 3 days, D-dimer increased at 1 to 4 weeks, and thrombocytosis was observed at 2 weeks.

**Conclusions:**

FVIII is elevated in most patients with CTEPH. After PEA, early but transient elevation of FVIII and fibrinogen, and delayed reactive thrombocytosis, occurs, and warrants careful postoperative anticoagulation to prevent thromboembolism recurrence.

## Introduction

Chronic thromboembolic pulmonary hypertension (CTEPH) as the disease spectrum of pulmonary hypertension (PH) is characterized by organized pulmonary arterial thrombus and fibrotic vascular remodeling.^1^ Incompletely resolved acute or recurrent pulmonary embolism (PE) may lead to CTEPH (pooled incidence of 3.4%^2^), but evidence of antecedent acute PE is often lacking.^1,2^ While the serious nature of the condition leads to progressive PH and right heart failure, pulmonary endarterectomy (PEA), together with indefinite anticoagulation, is the main and potentially curative treatment modality for patients eligible for surgery.^1^ Balloon pulmonary angioplasty and PH targeted medical therapy complement the management of patients with CTEPH.^1^

Multiple scientific efforts have been made to understand the etiology and risk factors of CTEPH .^3,4^ With respect to inherited and acquired thrombophilias and other coagulation abnormalities, coagulation factor VIII (FVIII) and its carrier protein von Willebrand factor (VWF) are constantly elevated before and 1 year after PEA surgery.^5,6^ Antiphospholipid antibodies are up to 20% prevalent in the disease^7^ whereas other classical thrombophilias do not seem to contribute to CTEPH.^7^ Altered fibrin structure and fibrinolysis^8,9^ have been observed and platelets of patients with CTEPH are activated and also hyper-responsive.^10^

Inflammation and coagulation have a multifold reciprocal relationship, called thromboinflammation, and tissue damage related to surgery propagates the inflammatory response.^11^ Reports on coagulation biomarkers after major surgery illustrate that elective abdominal surgery itself increases coagulation activity, including fibrinogen and FVIII levels early on.^12^ In addition, fibrinogen, FVIII and FIX values increased 24 h after coronary artery bypass surgery, whereas the levels of FVII, FXI, and FXIII decreased.^13^ Although it is described that FVIII and fibrinogen are elevated during the first day after PEA,^14^ to our knowledge, longitudinal characterization of the coagulation biomarker evolution in this prothrombotic patient group is missing. We endeavored to provide a broader understanding of the coagulation profile after PEA, which could be important for optimal postoperative anticoagulation management and could have implications for CTEPH pathogenesis.

## Methods

### Study Design

We conducted a single-center (Helsinki University Hospital, the national center for PEA surgery in Finland) observational study with an objective to characterize the coagulation biomarker profiles during the initial preoperative and extended postoperative (till 3 months) phase in patients undergoing PEA surgery. We included 17 consecutive patients operated between November 2020 and May 2022. Perioperative coagulation biomarkers were determined up to 12 months, and postoperative FVIII levels were compared with other coagulation biomarkers. We collected all related data from local electronic medical and laboratory systems (Epic Apotti, Weblab Clinical). The study was reviewed and approved by The Institutional Review Board of the Helsinki University Hospital, Helsinki, Finland (HUS/237/2021).

### Laboratory Measurements

The following coagulation assays (reagent and unit in parenthesis) in citrated (3.2%) plasma were analyzed at the local Central Coagulation Laboratory (HUSLAB of Helsinki University Hospital): FVIII (FVIII:C one-stage clotting assay [IU/dl], pathromtin SL and FVIII deficient plasma), fibrinogen (Clauss method [g/l], HemosIL Q.F.A.Thrombin, Werfen, Barcelona, Spain; D-dimer [mg/l] HemosIL D-Dimer HS 500), antithrombin (AT [%], a chromogenic assay Berichrom Antithrombin III), thrombin time ([s], BC Thrombin reagent, Siemens), activated partial thromboplastin time (APTT [s], Actin FSL®, Siemens) and anti-FXa activity (anti-FXa [IU/ml], HemosIL Liquis Anti-Xa, Mediq Suomi Oy). We acquired data of these coagulation markers preoperatively and from the days 1, 2, 3, 7, 14, 30, 90, and 12 months after the operation, if available.

In addition, we measured the dynamics of white blood cell (WBC) count, C-reactive protein (CRP, mg/l), and platelet count (10^9^/l) from the same time points. Preoperative plasma values of prothrombin time (Medirox Owren's PT [%] Medirox, Nyköping, Sweden), FXIII (F-XIII, %), VWF antigen (VWF:Ag, %) and VWF glycoprotein GPIb binding activity (VWF:Act, %), homocysteine (Hcyst, µmol/l), low-density lipoprotein (mmol/l), and triglycerides (Trigly, mmol/l) were collected. Additionally, patients were screened for protein C and S deficiencies, antiphospholipid antibodies as well as Factor V Leiden and FII G20210A mutations.

### Perioperative Antithrombotic Treatment

Before PEA, 8 patients were anticoagulated with warfarin, 6 with LMWH, 2 with fondaparinux and 1 with unfractionated heparin (UFH), and due to the thrombotic burden, 15 of 17 patients also received low-dose aspirin (ASA, 100 mg) and statins. ASA was withheld for 5 days prior to PEA. Postoperative anticoagulation was initiated by UFH (1 patient) or LMWH (dalteparin in 8, enoxaparin in 7, and tinzaparin in 1 patient) at 372 ± 177 min after surgery and ASA 2.8 ± 1.8 days after the operation. LMWH dosage was titrated until the targeted through anti-FXa activity of 0.2-0.5 IU/ml was achieved and switched to fondaparinux before discharge in 2 patients. At 3 months, 14 patients were treated with LMWH (9 with dalteparin and 5 with enoxaparin) and 2 patients with fondaparinux, and 16 patients received ASA. The long-term antithrombotic care and the switch to oral anticoagulation with warfarin was later determined on individual basis.

### Intraoperative Surgical, Anesthesia, and Transfusion Management

PEA was performed from median sternotomy, the patient was cooled to 18°C to 20°C using cardiopulmonary bypass (CBP), and bilateral PEA was performed under deep hypothermic circulatory arrest. Unfractionated heparin (Leo Pharmaceutical Products, Denmark) was used for intraoperative anticoagulation monitored by activated clotting time (ACT) (target > 480 s Kaolin-ACT, Medtronic.Inc. ACTII, Minneapolis, MN, USA). Before the initiation of CBP, 500 to 1000 ml of blood was harvested, and returned to the patient after weaning off CPB, heparin reversal by protamine sulfate, and decannulation. During CPB to maintain patients’ volume status and to minimize the use of crystalloids (plasmalyte 50 mg/ml, Baxter) and possible volume overload autologous blood transfusion (cell saver), allogenic red blood cell (RBC) transfusions (Hb < 60 g/l), 2 to 6 units of solvent-detergent treated standardized plasma (Octaplas®, Octapharma AG, Lachen, Switzerland) or albumin 20% were used. Tranexamic acid was used 30 mg/kg intravenously before the surgical incision and again 15 mg/kg every 2 h for the duration of CPB. ACT was controlled every 20 min on CPB and 3 min after each heparin bolus. After CPB, administration of protamine and harvested blood infusion, coagulation status was controlled (heparinase-ACT, complete blood count, APTT, PT, fibrinogen, AT and D-dimer). Postoperatively in the operation room allogenic RBC were transfused if Hb < 90 g/l or Hct < 30%. The threshold for platelet transfusion was the platelet count <100 ×10^9^/l and for standardized plasma, Octaplas®, PT < 30%.

### Statistics

Continuous variables are expressed either as mean with standard deviation (by default) or median with interquartile range (ICU and hospital days) (Tables 1 and 2). Coagulation biomarker levels are reported as mean with standard deviation (SD). One-way analysis of variance (ANOVA) with Dunnet’s multiple comparisons test was used when longitudinally comparing preoperative to postoperative values or later postoperative values to the first postoperative day. Linear regression model and Pearson coefficient test were applied when assessing correlations between preoperative and peak FVIII levels and preoperative and day 90 FVIII levels. Statistical analysis and data visualization were performed by GraphPad Prism 9.3.1 (GraphPad Software, Inc).

**Table 1.** Baseline Characteristics.

Variable	n = 17
Age, years	50 ± 18
Sex, female	6 (35%)
Body mass index, kg/m^2^	28 ± 6
Positive smoking status (current or former)	5 (30%)
ABO blood group (O 33% in Finland)	
A	10 (59%)
B	2 (12%)
AB	0 (0%)
O	5 (29%)
No thrombophilia	4 (24%)
Any thrombophilia	13 (76%)
Elevated FVIII	12 (71%)
Without other thrombophilias	7 (41%)
With Factor V Leiden	1 (6%)
With essential thrombocytosis and Factor V Leiden	1 (6%)
With antiphospholipid syndrome	3 (18%)
Normal FVIII	
With antiphospholipid syndrome	1 (6%)
History of acute pulmonary embolism	13 (76%)
History of deep vein thrombosis	7 (41%)
Performance status (NYHA class)	
NYHA I	0 (0%)
NYHA II	8 (47%)
NYHA III	8 (47%)
NYHA IV	1 (6%)

Abbreviations: FVIII, coagulation factor VIII; NYHA, New York Heart Association.

**Table 2.** Perioperative, Hemodynamic, and Surgical Results.

	n = 17
CTEPH disease level	
1 = main	5 (29%)
2 = lobar	4 (24%)
3 = segmental	8 (47%)
4 = subsegmental	0 (0%)
Perioperative PVR, WU	
Pre	9.2 ± 4.9
Post	4.2 ± 2.2
Last at ICU	3.2 ± 1.7
Perioperative mPAP, mm Hg	
Pre	48 ± 19
Post	31 ± 10
Last at ICU	25 ± 4.9
Intraoperative transfusions	
RBC, units	2.5 ± 2.3
Platelets, units	2.9 ± 1.2
FFP, 200 ml doses	4.8 ± 1.3
Postoperative transfusions	
RBC, units	7 (41%), 3.4 ± 2.3
Platelets, units	2 (12%), 2 ± 0
FFP, 200 ml doses	1(6%), 1 ± 0
Thrombotic complications*	1 (10%)
Major bleeding**	1 (10%)
Minor bleeding***	2 (20%)
ICU stay, days (median)	4 (IQR = 6)
Hospital stay, days (median)	13(IQR = 9)
3 months survival	94%
3 months NYHA 1 performance	12 (70%)

*Heparin-induced thrombocytopenia.

**Hemopericardium.

***Femoral pseudoaneurysms.

Abbreviaitons: CTEPH, chronic thromboembolic pulmonary hypertension; FFP, fresh frozen plasma; ICU, intensive care unit; IQR, interquartile range; mPAP, mean pulmonary artery pressure; NYHA, New York Heart Association; PVR,  pulmonary vascular resistance; RBC, red blood cell; WU, Wood Units.

## Results

### Baseline Characteristics

Patients were middle-aged (50 ± 18 years), nonsmokers and meanly with minor obesity (Table 1). History of active malignancy or splenectomy was absent, and 2 patients were on thyroid replacement therapy for hypothyroidism. Prevalence of baseline thrombophilia was 76%, and history of acute PE was frequent (76%). The disease burden of CTEPH was substantial since 53% of the patients were classified having NYHA III or IV performance status and the mean pulmonary artery pressure was 48 ± 19 mm Hg before PEA. Eight patients were bridged to surgery with PH-targeted medication (riociguat with or without macitentan).

### Perioperative, Hemodynamic, and Surgical Results

PEA significantly improved hemodynamics and symptoms (Table 2). Regarding postoperative blood product transfusions, 7 patients received allogenic RBC during the hospital stay. Thrombotic complications occurred in a single patient who developed postoperative heparin-induced thrombocytopenia, and a new-onset femoral vein thrombosis, and anticoagulation was subsequently switched from LMWH to fondaparinux. One patient required surgical sub-xiphoidal pericardial effusion drainage at 19 days after PEA, and 2 patients developed femoral artery pseudoaneurysms at the sites of arterial lines and were successfully treated with ultrasound-guided local thrombin injections. After median ICU stay of 4 days and 13 total hospital days, 16 of 17 patients were discharged. One of the patients developed postoperative cholecystitis requiring laparotomy, but later died of bacteremia and multi-organ failure. Most patients (70%) had improved performance status to NYHA I at 3 months.

### Baseline Laboratory Data

General biomarkers did not identify any renal or liver dysfunction or baseline inflammation (Table 3). Most patients were on lipid lowering therapy and hyperlipidemia was well controlled. Plasma N-terminal prohormone B-type natriuretic peptide (NT-ProBNP) was markedly elevated (1350 ± 1420 ng/l). Baseline coagulation assays revealed elevated FVIII in 70% of the patients, mean being 215 ± 67 IU/dl. Although mean VWF:Ag and VWF:Act levels were borderline, the same patients with elevated FVIII levels had also VWF:Ag and VWF:Act levels above the respective upper normal limits (190%). Homocysteine and APTT baseline levels were marginally elevated while there were no deviations in PT, fibrinogen, antithrombin, FXIII, or D-dimer values. Characterization of baseline thrombophilias (elevated FVIII, Factor V Leiden, antiphospholipid syndrome and essential thrombocytemia) are found in Table 1.

**Table 3.** Baseline Laboratory Results.

Variable (reference range and unit)	Mean with SD (n = 17)
Platelet count (150-360 E9/l)	220 ± 64
WBC count (3.4-8.2 E9/l)	7.7 ± 2.8
C-reactive protein (<4 mg/l)	8.3 ± 14
Creatinine (60-100 µmol/l in men, 50-90 µmol/l in women)	82 ± 16
Bilirubin (<20 µmol/l)	23 ± 11
ProBNP (<84-352 ng/l)	1350 ± 1420
LDL (<3.0 mmol/l)	2.3 ± 1.1
Triglycerides (<1.7 mmol/l)	1.5 ± 0.48
Homocysteine (<15 µmol/l)	15 ± 7.1
APTT (28-37 s)	38 ± 6.8
PT (70%-130%)	65 ± 30
Fibrinogen (2-4 g/l)	3.1 ± 0.8
D-dimer (<0.5 mg/l)	0.7 ± 2.0
Antithrombin (85%-125%)	94 ± 16
VWF:Ag (50%-190%)	206 ± 59
VWF activity (50%-190%)	186 ± 65
FVIII (60-160 IU/dl)	215 ± 67
Elevated FVIII (%)	12 (70%)
FXIII (76%-156%)	100 ± 36

Abbreviations: APTT, activated partial thromboplastin time; FVIII, coagulation factor VIII; LDL, low-density lipoprotein; PT, prothrombin time; VWF, von Willebrand factor; WBC, white blood cell.

### FVIII is Upregulated After PEA

**Figure 1.**
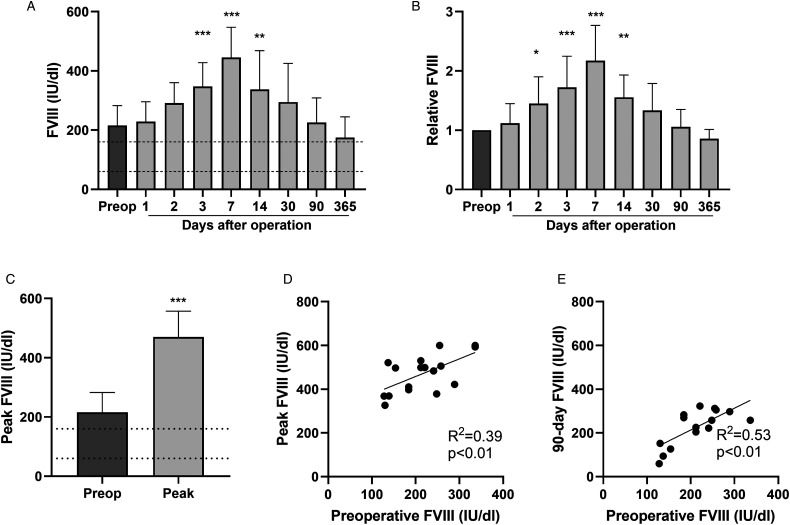
FVIII levels after PEA. Preoperative baseline and postoperative FVIII levels were determined from 17 patients undergoing PEA (n = 10-17 per time point). FVIII levels at baseline, and 1, 2, 3, 7, 14, 30, 90, and 365 days after PEA (A). FVIII levels relative to the baseline levels (B). Preoperative and peak postoperative FVIII levels (C). Preoperative FVIII levels were compared with the peak postoperative (D) or 3-month levels (E) of the respective patients. Data are expressed as mean ± SD and analyzed by one-way ANOVA with Dunnett's multiple comparisons test comparing postoperative values to preoperative values (A and B), student's t test (C), and by linear regression and Pearson coefficient test (D and E). Dashed lines indicate the range (A and C) of normal values. *P < .05, **P < .01, ***P < .001. Abbreviations: ANOVA, analysis of variance; FVIII, coagulation factor VIII; PEA, pulmonary endarterectomy.

After surgery, FVIII levels gradually elevated up to 2-fold at the seventh postoperative day followed by a slow return to the baseline level (Figure 1A and B). Peak postoperative FVIII levels reached 471 ± 87 IU/dl and positively correlated with the respective baseline levels (Figure 1C and D). A strong correlation was found between the baseline and 3-month FVIII levels (Figure 1E), indicating that after the initial postoperative elevation of FVIII, the patients reverted to their individual baseline.

### Other Coagulation Biomarkers After PEA

**Figure 2.**
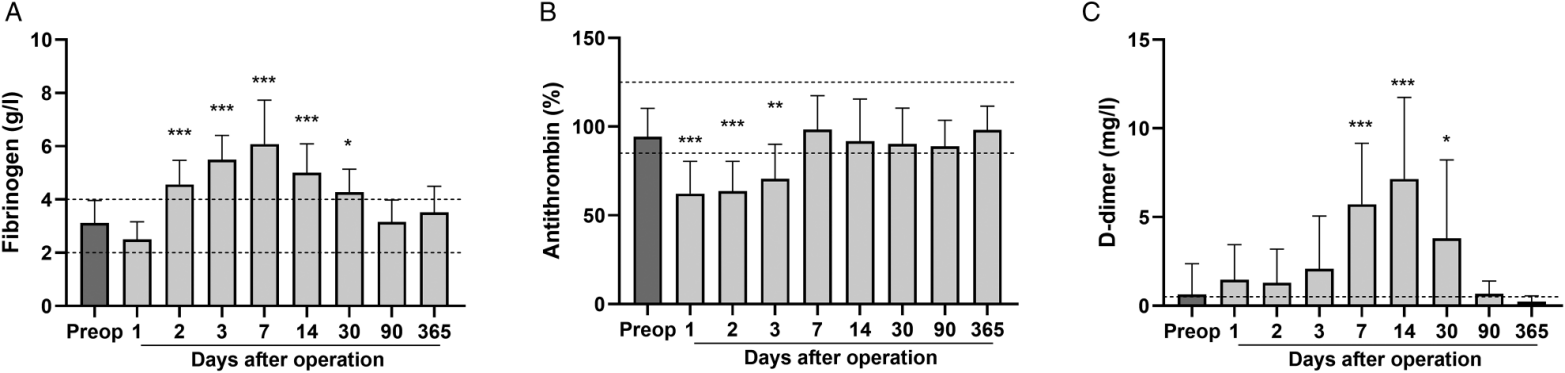
Fibrinogen, antithrombin, and D-dimer levels after PEA. Preoperative and postoperative coagulation biomarkers were determined from 17 patients undergoing PEA (n = 10-17 per time point). Fibrinogen (A), antithrombin (B) and D-dimer (C) levels before operation, and 1, 2, 3, 7, 14, 30, 90, and 365 days after PEA. Data are expressed as mean ± SD and analyzed by one-way ANOVA with Dunnett's multiple comparisons test comparing postoperative values to preoperative levels. Dashed lines indicate the range (A and B) and the upper limit (C) of normal values. *P < .05, **P < .001, ***P < .001. Abbreviations: ANOVA, analysis of variance; PEA, pulmonary endarterectomy.

**Figure 3.**
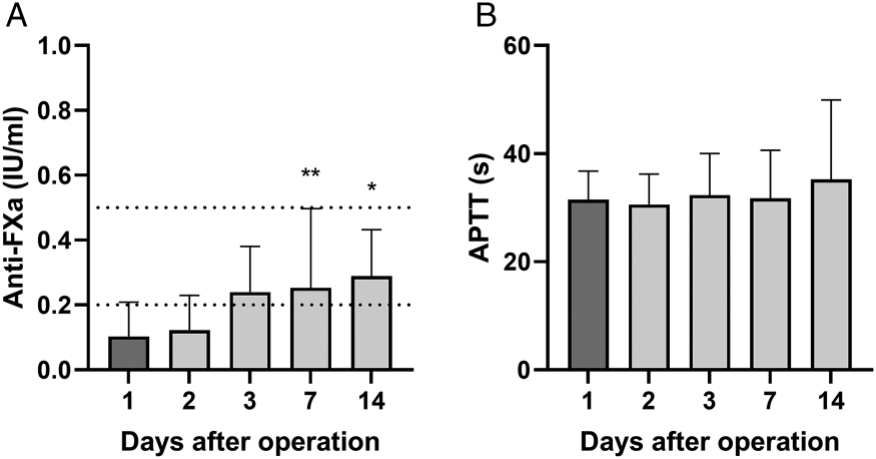
Coagulation assays after PEA. Subcutaneous twice daily low-molecular-heparin was used for postoperative anticoagulation starting at 4 to 6 h after surgery, and gradually titrated to therapeutic anti-FXa 12 h trough levels of 0.2 to 0.5 IU/ml. Anti-FXa (A) and APTT (B) at 1, 2, 3, 7, and 14 days after PEA (n = 12-17 per time point). Data are expressed as mean ± SD and analyzed by one-way ANOVA with Dunnett's multiple comparisons test comparing values to day 1 levels. Dashed lines indicate the therapeutic target range for 12 h anti-FXa (A). *P < .05, **P < .01. Abbreviations: ANOVA, analysis of variance; APTT, activated partial thromboplastin time; PEA, pulmonary endarterectomy.

Fibrinogen levels (reference 2-4 g/l) were normal before surgery but transiently increased after surgery, resembling the postoperative FVIII upregulation (Figure 2A). Antithrombin (AT, 85%-125%) levels decreased during the first postoperative days followed by a rapid recovery already within the first week (Figure 2B). D-dimer (<0.5 mg/l), an indicator of fibrin turnover, markedly peaked at the second postoperative week with subsequent gradual normalization (Figure 2C). Mean 12 h anti-FXa levels reached therapeutic levels (0.2-0.5 IU/ml) by the third postoperative day following the gradual increase of postoperative LMWH dosing (Figure 3). APTT and thrombin time values remained constant during the postoperative period.

### Acute Phase Reaction After PEA

CRP was normal (<4 mg/l) before the surgery, but the levels of this acute phase protein transiently elevated after surgery peaking at day 2 (171 ± 45 mg/l, *P* < .0001) and returned to low levels by 2 weeks (Supplemental Figure 1A). The mean WBC count remained normal during the whole perioperative period (Supplemental Figure 1B). Normal preoperative platelet count (220 ± 64×10^9^/l, reference range 150-360×10^9^/l) was followed by a transient initial postoperative decline (days 2-3), and a delayed rise (494 ± 159 ×0^9^/l, *P* < .0001) at 2 weeks (Supplemental Figure 1C).

### Correlation of Postoperative FVIII With Other Coagulation Biomarkers

**Figure 4.**
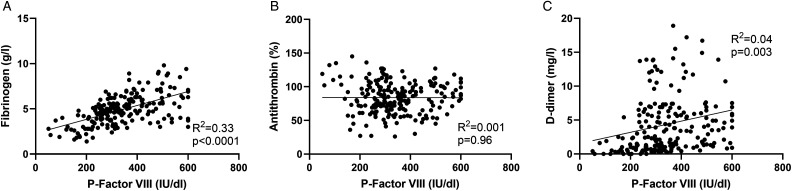
Correlation of FVIII levels with other coagulation biomarkers after PEA. The 207 postoperative samples of 17 patients up to 90 days after PEA were analyzed, and FVIII levels were compared with respective fibrinogen (A), antithrombin (B), and D-dimer values (C). Data were analyzed by linear regression and Pearson coefficient test. Abbreviations: PEA, pulmonary endarterectomy; FVIII, coagulation factor VIII.

Additionally, we analyzed correlations between FVIII levels and other coagulation biomarkers before surgery, and after PEA (Figure 4). The baseline FVIII positively correlated with fibrinogen, and VWF:Ag and VWF:Act, whereas no significant correlation was found with AT or FXIII (Supplemental Figure 2). After PEA, FVIII positively correlated with fibrinogen, and negatively with APTT, whereas correlation with anti-FXa, AT or D-dimer was absent (Figure 4 and Supplemental Figure 3).

## Discussion

Along with anticoagulation, PEA remains the main treatment for patients with CTEPH eligible for surgery and it also improved the hemodynamics and symptoms in our current study cohort. Interestingly, temporal changes in different coagulation biomarkers were observed after PEA, which may reflect both the distinct prothrombotic state of patients with CTEPH, and the acute phase reaction related to surgery. Most notably, the levels of the concentration-dependently prothrombotic^15^ FVIII were elevated already at baseline, and a profound but transient FVIII elevation together with fibrinogen was detected after surgery. These findings may have clinical implications when making decisions on optimal postoperative anticoagulation management after PEA. In addition, our finding of a pronounced prothrombotic acute phase response in patients with CTEPH may give insight to the pathogenesis underlying this thrombotic disease.

Consistent with the risk factors for CTEPH,^5,7^ most patients in our study had underlying hypercoagulable states. Although these included cases of Factor V Leiden, antiphospholipid syndrome and essential thrombocytosis, the most common prothrombotic condition was elevated FVIII. 12 patients (71%) had FVIII levels above the reference range (60-160 IU/dl) before the PEA, and the mean baseline FVIII level was 216 ± 67 UI/dl. This aligns with the original findings by Bonderman et al.^5^ Our present study thus further emphasizes the role of elevated FVIII in CTEPH. With respect to preoperative FVIII levels in other elective surgeries, patients undergoing major abdominal or gynecological surgery had baseline FVIII levels in the normal range.^12,16^ Preoperative FVIII was elevated in 35% of patients with elective coronary artery bypass graft (CABG) in the study of Ternström et al,^13^ and the mean baseline FVIII was within the normal range in patients undergoing various cardiac surgeries.^17^

The most striking finding of our study was the marked FVIII elevation after PEA. Although previous reports indicate that PEA upregulates FVIII 1 to 3 days after surgery,^14^ and that FVIII levels revert to baseline levels 1 year after successful PEA,^5^ our study provides details on the temporal FVIII changes after surgery. FVIII levels peaked 1 week after PEA and then gradually returned to the baseline values by 3 months. This pattern is most likely related to the acute phase reaction associated with PEA and is also observed in other major surgeries. After elective major abdominal surgery, FVIII levels significantly raised (peak FVIII activity levels <250%) and stayed elevated in the 10 postoperative days study period.^12^ FVIII levels 24 h after CABG were significantly elevated (263 ± 76%) .^13^ Edelman et al showed that CABG results in a hypercoagulable state, and that the overall hemostatic potential is more pronounced in patients with on-pump CABG compared to patients with off-pump CABG.^18^ Collectively, these findings indicate that in addition to the surgical trauma, the use of CPB also affects the postoperative coagulation profile. All in all, the combination of remarkable baseline thrombogenicity and profound acute phase prothrombotic reaction as presented in our data is distinctive in patients with CTEPH.

We found that the patients with the highest baseline FVIII levels also had the highest peak levels, and the levels reverted to their individual baseline at 3 months. It seems that the patients with low or high FVIII baseline levels have their unique constitutive FVIII production rate, which is further amplified by the acute phase response. The genetic and molecular mechanisms that regulate production of FVIII are not completely understood.^15^ However, it is known that the proinflammatory transcription factor nuclear factor kappa B (NF-κB) contributes to FVIII production^19^ and that proinflammatory factors interleukin-1 (IL-1) and IL-6^20,21^ increase the levels of circulating FVIII and its carrier protein VWF.^15^ Interestingly, epigenetic modifications and increased NF-κB2 binding in the VWF promoter drive VWF transcription and may link inflammation and thrombosis in patients with CTEPH.^22^ However, it remains to be proven whether similar molecular mechanisms are involved in FVIII regulation.

In addition to the elevated FVIII levels, PEA also resulted in divergent changes in other coagulation biomarkers. Fibrinogen levels were increased, and positively correlated with the FVIII levels, which indicates similar acute phase responses for these 2 factors and emphasizes the postoperative thrombotic state of patients with CTEPH.^23^ Elevation of postoperative fibrinogen levels are also reported in other major cardiac and noncardiac surgeries.^13,24^ In contrast, an early AT decline was observed which likely reflects early intraoperative thrombin generation and consumption of AT. We also detected a significant delayed rise of fibrin turnover (D-dimer) at 1 to 4 weeks after surgery. This again may mirror the hypercoagulable state, or potentially the re-endothelization process that may follow the surgical removal of the native endothelial layer of the pulmonary artery,^25^ and fibrinolysis occurring at tissue level in the microcirculation. Long-lasting elevation of D-dimer is also seen in the setting of CABG.^26^

Interestingly, a distinct postoperative platelet count pattern was also observed. Platelet counts decreased during the first days after surgery, potentially due to intraoperative consumption, and possible type 1 direct heparin effects ^27^ but increased above the normal reference range (to 494 ± 159×10^9^/l) 2 weeks after surgery. Similar delayed thrombocytosis has been reported after coronary bypass surgery.^28^ IL-6-mediated acute phase stimulation of thrombopoietin could be involved.^29^ As platelets of patients with CTEPH are highly active,^10^ the reactive postoperative thrombocytosis may further aggravate the thrombotic state.

Indefinite anticoagulation is essential in CTEPH. Conventionally and preferably, anticoagulation in patients with CTEPH is achieved with a vitamin K antagonist but also direct oral anticoagulants may be used in patients without an antiphospholipid syndrome.^30^ Alongside anticoagulation, all our patients received low-dose aspirin to target highly activated platelets,^10^ and statins due to their potential pleiotropic effects on endothelial protection, coagulation, and fibrinolysis.^31^ Balancing adequate anticoagulation is especially important after PEA to prevent thrombotic complications, especially pulmonary artery re-thrombosis that might compromise the long-term surgical outcomes, but equally to avoid postoperative bleeding, and unfractionated heparin and LMWH are common options for the immediate postoperative anticoagulation. Evidence-based optimal anticoagulation strategy for initial postoperative era after PEA (including timing of peroral anticoagulation initiation) is lacking as far as we know. Heparin products have several pleiotropic anti-inflammatory effects that are potentially favorable during the acute prothrombotic phase.^32^ In addition to anticoagulant, the choice of anticoagulation monitoring may be equally important, as it may be affected by acute phase proteins and prominent coagulation activity.^33^ We chose to use anti-FXa activity levels to guide the postoperative anticoagulation, as APTT is shortened by elevated FVIII and result in (pseudo) heparin resistance (Supplemental Figure 3).^14^

Our study has several limitations. It is retrospective, the sample size is small, and some data points were missing. Additionally, we did not have a comparative non-PEA cohort in order to parallel our coagulation biomarker results to other patient groups.

In conclusion, our data support the prior association of elevated FVIII in CTEPH. In addition, we show that PEA results in a prothrombotic state with a profound elevation of FVIII and fibrinogen, and a delayed reactive thrombocytosis. These acute phase changes on top of the thrombogenic baseline properties may contribute the pathogenesis of CTEPH and have clinical implications on optimal anticoagulation management after first weeks of PEA.

## Supplemental Material

sj-docx-1-cat-10.1177_10760296231158369 - Supplemental material for Upregulation of Coagulation Factor VIII and Fibrinogen After Pulmonary Endarterectomy in Patients with Chronic Thromboembolic Pulmonary Hypertension Click here for additional data file. Supplemental material, sj-docx-1-cat-10.1177_10760296231158369 for Upregulation of Coagulation Factor VIII and Fibrinogen After Pulmonary Endarterectomy in Patients with Chronic Thromboembolic Pulmonary Hypertension by Lasse Myllylahti, MD, Jussi Ropponen, MD, PhD, Mikko Lax, MD, Riitta Lassila, MD, PhD, and Antti I. Nykänen, MD, PhD in Clinical and Applied Thrombosis/Hemostasis

## Abbreviations

CTEPH: chronic thromboembolic pulmonary hypertension
PH: pulmonary hypertension
PEA: pulmonary endarterectomy
LMWH: low-molecular-weight heparin
FVIII: coagulation factor VIII
