# Supplementary material for: Upregulation of Coagulation Factor VIII and Fibrinogen After Pulmonary Endarterectomy in Patients with Chronic Thromboembolic Pulmonary Hypertension
Source: Clin Appl Thromb Hemost. 2023 Mar 8;29:10760296231158369. doi: 10.1177/10760296231158369 (PMC9998419; doi:10.1177/10760296231158369)

**ssSUPPLEMENTARY FIGURE LEGENDS**

**Supplementary Figure 1. C-reactive protein levels, WBC and platelet counts after pulmonary endarterectomy.** Pre- and postoperative CRP levels (A), WBC (B) and platelet counts (C) were determined from 17 patients undergoing PEA. Data are expressed as mean±SD and analyzed by One-way ANOVA with Dunnett's multiple comparisons test comparing postoperative values to preoperative levels. Dashed lines indicate the upper limit (A) and range (B-C) of normal values. *p<0.05, **p<0.01, ***p<0.001.

**
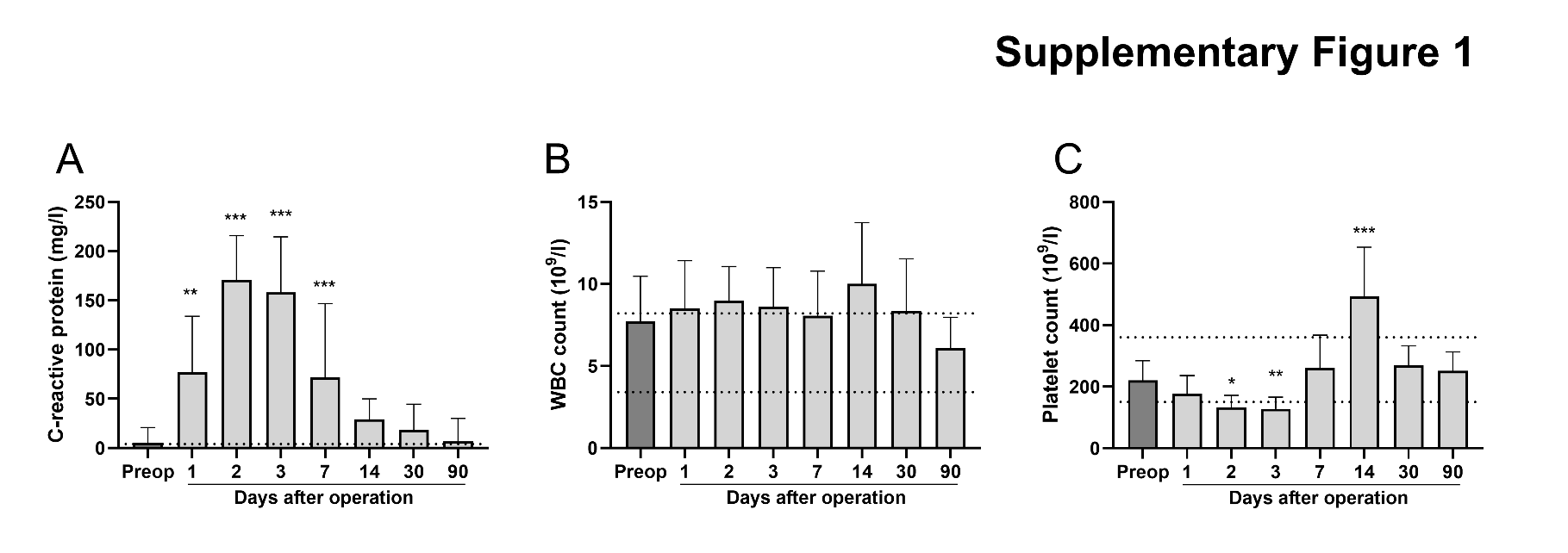
**

**Supplementary Figure 2. Correlation of preoperative FVIII levels with coagulation biomarkers before pulmonary endarterectomy.** Correlations of preoperative FVIII levels with the respective preoperative fibrinogen (A), antithrombin (B), FXIII (C), VWF:Ag (D) and VWF:Act values (E) were assessed. Data were analyzed by linear regression and Pearson coefficient test.


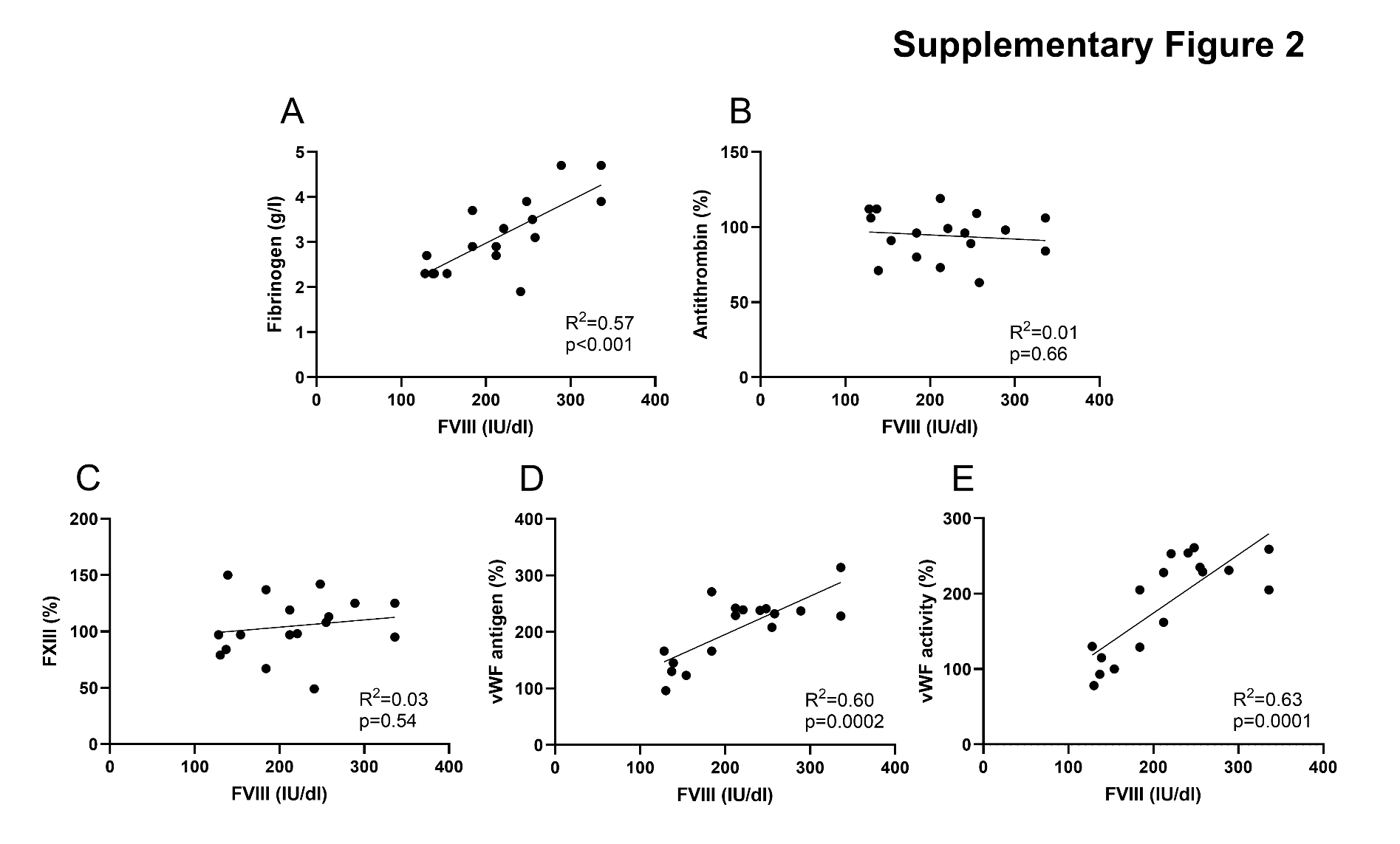


**Supplementary Figure 3.** Correlation of FVIII levels with coagulation assays after pulmonary endarterectomy. 207 postoperative samples of 17 patients up to 90 days after pulmonary endarterectomy were analysed, and correlations of FVIII levels with respective anti-FXa activity (A) and APTT (B) values were assessed. Data were analyzed by linear regression and Pearson coefficient test.


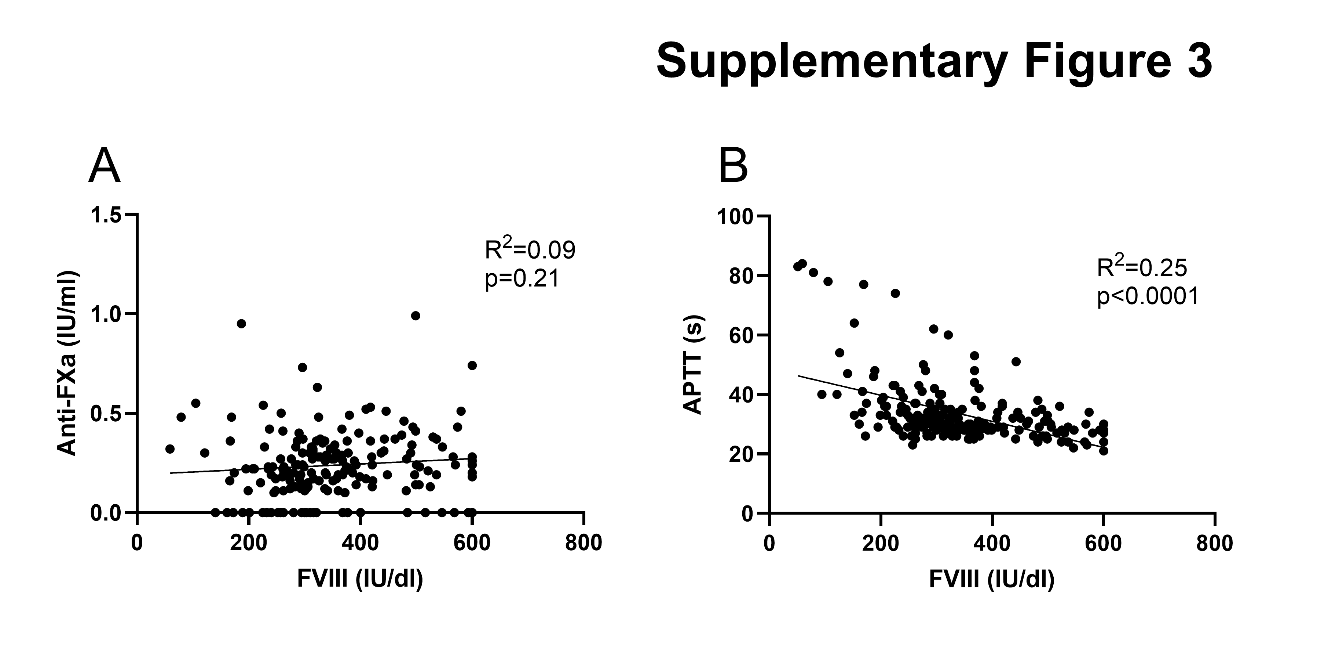

Supplement: sj-docx-1-cat-10.1177_10760296231158369 - Supplemental material for Upregulation of Coagulation Factor VIII and Fibrinogen After Pulmonary Endarterectomy in Patients with Chronic Thromboembolic Pulmonary Hypertension [file sj-docx-1-cat-10.1177_10760296231158369.docx]
